# The Dynamics of the Human Leukocyte Antigen Head Domain Modulates Its Recognition by the T-Cell Receptor

**DOI:** 10.1371/journal.pone.0154219

**Published:** 2016-04-28

**Authors:** Estefanía García-Guerrero, José Antonio Pérez-Simón, Luis Ignacio Sánchez-Abarca, Irene Díaz-Moreno, Miguel A. De la Rosa, Antonio Díaz-Quintana

**Affiliations:** 1 Instituto de Biomedicina de Sevilla (IBIS)/Hospital Universitario Virgen del Rocío/CSIC/Universidad de Sevilla, Seville, Spain; 2 Hospital Universitario de Salamanca/Servicio de Hematología, Salamanca, Spain; 3 Instituto de Bioquímica Vegetal y Fotosíntesis, cicCartuja, Universidad de Sevilla—CSIC, Seville, Spain; Oak Ridge National Laboratory, UNITED STATES

## Abstract

Generating the immune response requires the discrimination of peptides presented by the human leukocyte antigen complex (HLA) through the T-cell receptor (TCR). However, how a single amino acid substitution in the antigen bonded to HLA affects the response of T cells remains uncertain. Hence, we used molecular dynamics computations to analyze the molecular interactions between peptides, HLA and TCR. We compared immunologically reactive complexes with non-reactive and weakly reactive complexes. MD trajectories were produced to simulate the behavior of isolated components of the various p-HLA-TCR complexes. Analysis of the fluctuations showed that p-HLA binding barely restrains TCR motions, and mainly affects the CDR3 loops. Conversely, inactive p-HLA complexes displayed significant drop in their dynamics when compared with its free versus ternary forms (p-HLA-TCR). In agreement, the free non-reactive p-HLA complexes showed a lower amount of salt bridges than the responsive ones. This resulted in differences between the electrostatic potentials of reactive and inactive p-HLA species and larger vibrational entropies in non-elicitor complexes. Analysis of the ternary p-HLA-TCR complexes also revealed a larger number of salt bridges in the responsive complexes. To summarize, our computations indicate that the affinity of each p-HLA complex towards TCR is intimately linked to both, the dynamics of its free species and its ability to form specific intermolecular salt-bridges in the ternary complexes. Of outstanding interest is the emerging concept of antigen reactivity involving its interplay with the HLA head sidechain dynamics by rearranging its salt-bridges.

## Introduction

The specificity of the cellular immune response relies on cytotoxic T lymphocytes (CTLs) recognizing immunogenic oligopeptides presented in the context of MHC. The bio-molecular association of peptide-MHC (p-MHC) and TCR triggers CTL activation [1]. The T-cell receptor (TCR) must be able to discriminate between a huge number of alien antigens and self-peptides to avoid autoreactivity [2]. In the transplant context, T cells recognize either foreign major histocompatibility complexes (MHCs) or self-MHC proteins bound to exotic oligopeptides, generating an alloimmune response. The flexibility of free oligopeptides allows them to bind sites on the TCR surface different from the receptor site when not presented by MHC [3]. However, the specific chemical attribute that allows the MHC peptide-binding domain and the TCR antigen-binding site to discriminate self from non-self-peptides remains unclear [4].

The structure and conformation of a peptide in the HLA binding groove significantly affect the recognition of the p-HLA complex by the TCR [5–8]. In fact, altered peptide ligands (APL) carrying single amino acid substitutions trigger a response substantially different from that of the wild-type antigen notwithstanding the same carrier HLA and T-cell receptor participate in the recognition [5]. The molecular basis for such sensitive discrimination remains unclear. Indeed, various APLs may affect the TCR conformation in different ways or show distinct abilities modulate the dynamics of TCR oligomerization. In fact, Surface Plasmon Resonance analyses show that long p-MHC-TCR half-lives correlate tightly with agonist signals [6,7]. This scenario becomes more complex when we consider that TCRs recognition may be degenerated, the same receptor being able to bind MHC molecules carrying unrelated oligopeptides [8]. Moreover, the comparison of two human TCRs bound to the same p-HLA has shown that very different TCR sequences can recognize the same antigen, and that both induce full T cell activation [9].

To solve this puzzle demands to look at the atomic level. Several αβ TCR and TCR fragments have been crystallized in the unbound state [10–13] and bound to different p-MHC complexes of the Human Leukocyte Antigen (HLA) type [9,14–16]. The buried surface in these complexes is ca. 2,000 Å [16], in the verge between stable and transitory complexes [17]. This may provide the ability of modulating the lifetime of the complex by means of specific interactions [18]. Due to its long interaction range, electrostatics can be unspecific and lead to highly dynamic complexes [18–20]. However, residues with opposite charges at the rim of the binding interface may form salt-bridges, thereby stabilizing the complex and restraining its conformation dynamics [18]. For instance, mutations of charged residues that break single salt bridges impair Ferredoxin binding to both Nitrite Reductase and Glutamate Synthase [21]. Such interactions are detected in p-MHC-TCR complexes [8,9,22,23]. Alanine-scanning mutagenesis has shown the role of non-polar interactions in the p-MHC-TCR interface, also revealing the presence of π-cation interactions involving arginine and tyrosine residues [24]. Finally, the motions of residues at the complex interface suffer a restraint in their motions during the binding process. Hence, the conformation entropy of the binding partners in their free and bound states also modulates their association equilibrium [25–26].

**Fig 1:**
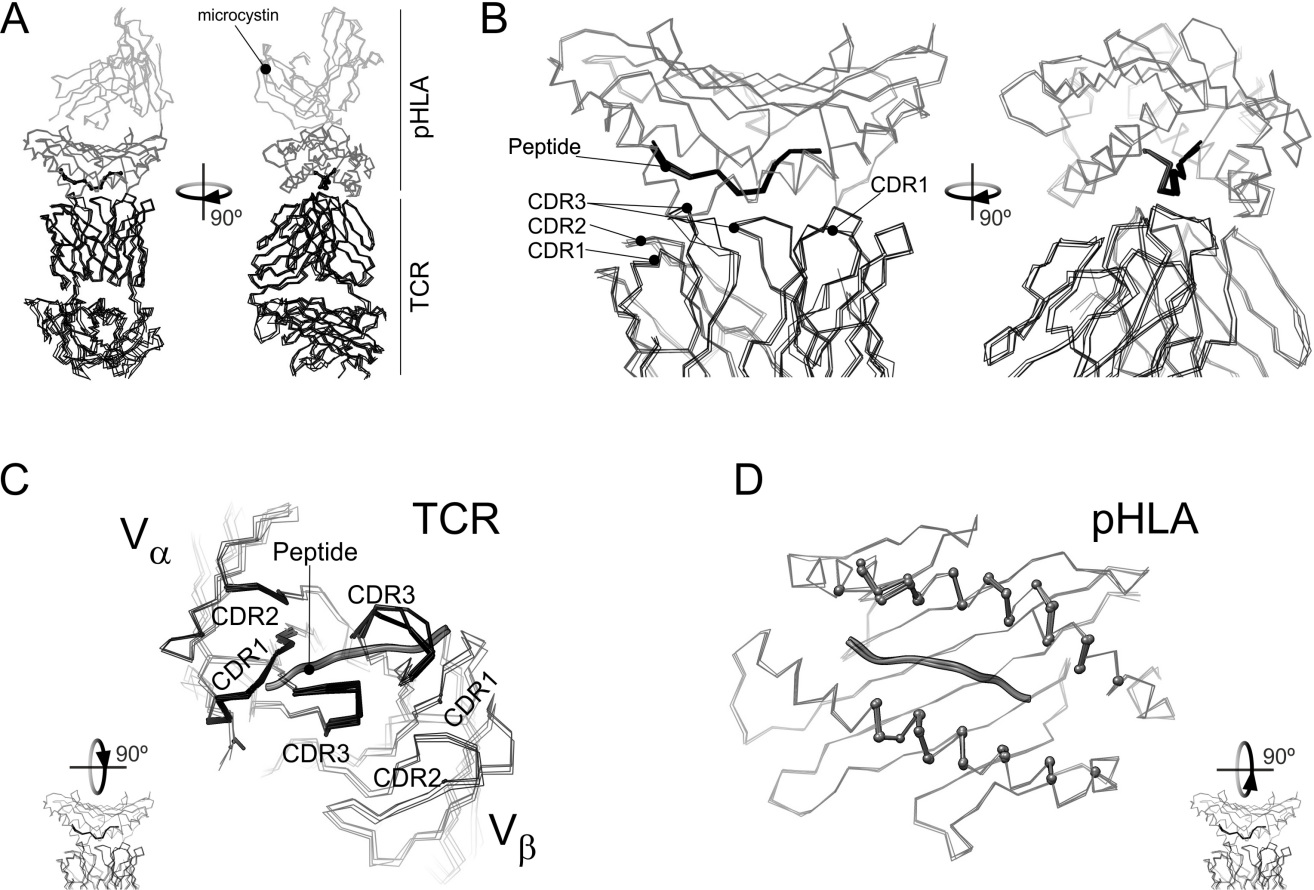
Overlay of the X-ray diffraction models for the WT and mutant TAX-HLA-TCR complexes. All the panels show an overlay of the Cα atoms of the TAX-HLA-TCR complex (pdb: 1ao7) [15] with those corresponding to V7R (pdb: 1qse) P6A (1qrn) and Y8A (pdb: 1qsf) [22]. A) Overlay of the full complex. TCR molecules are in black lines. Black ball-and-sticks represent the antigenic peptide. The HLA domain containing the peptide is in dark grey lines and the rest of the structure in light grey. B) detail of the ternary complex interface for the structure overlay in (A). C) Apical view of TCR and the antigenic peptide in the above overlay of the four structures. As in (A), TCR atoms are represented by lines, but those from residues closer than 6 Å from HLA are highlighted by stick representation. D) Apical view of HLA and the antigenic peptide for the four structures in the overlay. HLA residues closer than 6 Å from TCR are highlighted in a ball-and-stick representation.

The first structure of a human αβ TCR complex to be determined was that of the A6 TCR specific to the TAX oligopeptide (LLFGYPVYV) of the human T cell lymphotropic virus HTLV-1 bound to the human class I MHC molecule HLA-A2 [15]. The TAX peptide is a strong agonist that induces T-cell activation at very low concentrations. Single mutations on TAX inhibit T-cell function instead of triggering it [22]. The interactions of three peptide variants (P6A, V7R and Y8A) of Tax bound to HLA-A2 (HLA in this work) with the A6 T cell receptor (TCR in this work) have been extensively studied using T cell assays, kinetic and thermodynamic measurements, and XRD [9,22]. The V7R mutant weakly reacts and elicits the distinct cell responses at concentrations two orders of magnitude higher than TAX [22]. The P6A and V8A species can be considered unreactive, as they barely induce any response at concentrations several orders of magnitude higher than those at which the response to wild-type species saturates [22]. The three-dimensional structures of the three peptide-HLA-TCR complexes are remarkably similar to each other and to the wild-type agonist complex, besides minor changes at the interface to accommodate the peptide substitutions [22]. The backbone atoms for the four ternary complexes are overlaid in Fig 1, to show the high similarity between them. The tiny conformation differences found among various ternary complexes hardly correlate with T-cell responses, which may involve additional phenomena [23]. On the other hand, *in vitro* Surface Plasmon Resonance analyses of the recognition of TAX-based APLs correlate with the elicited responses *in cell* [23].

Surface Plasmon Resonance and X-ray diffraction (XRD) data indicate that the binding equilibrium is affected by the ability of the oligopeptide to rearrange during its recognition by TCR [23]. In addition, infrared spectroscopy indicates that very subtle changes of either the structure or the dynamics of the p-MHC complex modulate the affinity of the complex towards TCR [27, 28]. This knowledge led us to hypothesize that p-MHC plays a major role in T cell activation. However, XRD yields precise information about the conformation of the p-MHC-TCR complexes, rather than the dynamic features that modulate TCR recognition. Molecular dynamics (MD) provides such details at the atomic level of the interaction, giving valuable insights into the molecular basis of TCR recognition [29–34]. An early energy perturbation analysis, based on a 0.5 ns molecular dynamics (MD) trajectory performed on the p-MHC-TCR interface, showed that solvation effects are relevant as regards the stability of the complex [31]. These results provided a first link between XRD structure and the binding energy of a p-MHC-TCR complex.

In this work, we focus on the differences on the dynamic features of binary complexes (p-HLA) between HLA comprising each of four peptide variants (WT, P6A, V7R and Y8A) of Tax showing distinct degrees of T lymphocyte activation, and the corresponding ternary complex p-HLA-TCR [22]. With this purpose, we resorted to MD computations. Analysis of trajectories indicates that the various bimolecular complexes display distinct arrangement of surface salt bridges, resulting in slightly different dynamics of the complexes and changes in its electrostatics. In addition, we found differences between the patterns of interfacial salt bridges within the distinct complexes. This indicates that the properties of binary p-HLA species are substantially relevant as regards the discrimination between self and alien antigens. We show that changes in TAX affect strongly the binding entropy by perturbing HLA dynamics, which has a particular relevance. Moreover, our data suggests strongly that antigenicity of TAX species is linked to the ability of the peptide to elicit changes in the surface sidechains dynamics by shifting salt bridges in HLA.

## Methods

The published structures solved by XRD served as a starting point for this study. The HLA-A2/TAX/TCR-A6 system was chosen because it has been extensively studied experimentally, as have three peptide mutants (P6A, V7R and Y8A). The initial coordinates of the complexes were downloaded from Protein Data Bank [35] access codes: 1ao7 (TAX wild-type ternary complex; Garboczi *et al*., *1996*) [15, 36–38], 1qse (V7R-TAX; Ding *et al*., 1999) [22], 1qrn (P6A-TAX; Ding *et al*., 1999) [22, 39], 1qsf (Y8A-TAX, Ding *et al*., 1999) [22]. TAX (LLFGYPVYV) differs from the other peptides by single amino acid substitutions causing very different behaviors in T cells [22].

MD trajectories were calculated with the AMBER 9 package [40] in a DELL PowerEdge BeoWulf cluster under the AMBER-2003 force field [41]. Standard protocols were used to carry out the computations, and particular details were as described for similar calculations performed for the Human T cell intracellular antigen-1 [42]. Simulations were carried out under periodic boundary conditions in an orthorhombic cell solvated with TIP3P explicit water [43]. Particle Mesh Ewald summation cut-off for was 9 Å. A first energy minimization was performed on sidechains. Then, solvent was subjected to energy minimization followed by 300 ps NPT-MD computations. Temperature was regulated with Berendsen’s algorithm [44]. Then, for each protein, the whole system was energy minimized and submitted to 1 ns NVT-MD at 298 K, using 2.0 fs integration time steps for temperature equilibration. Production runs were computing under the microcanonical ensemble. Simulated production times are in Table 1. Snapshots were saved every ps. SHAKE algorithm [45] was used to constrain bonds involving hydrogen atoms. The trajectories were analyzed with the PTRAJ module of the AMBER package, MatLab 7.11 (MathWorks) and Origin8.5 (OriginLab).

**Table 1:** Summary of free HLA, free TCR and the various p-HLA and p-HLA-TCR complexes during MD trajectories.

Trajectory	Time of simulation (ns)	Average RMSD (Å)	Maximum RMSD (Å)	RMSD drift (pm ns^-1^)	<R_G_> (Å)
Free HLA	13.67	1.83	2.65	4	15.22
TAX-HLA	10.00	1.36	1.89	5	17.10
V7R-HLA	8.29	1.19	1.68	6	17.20
P6A-HLA	8.43	1.45	1.81	2	17.17
Y8A-HLA	8.27	1.47	2.26	10	17.26
Free TCR	10.00	1.84	2.65	6	23.85
TAX-HLA-TCR	9.50	1.33	1.73	1	30.17
V7R-HLA-TCR	10.00	1.81	2.76	7	30.43
P6A-HLA-TCR	9.50	1.94	2.95	4	30.66
Y8A-HLA-TCR	10.00	1.61	2.60	6	30.37

HLA and TCR stand for the HLA-A2 and TCR-A6 species. RMSD means root mean square deviation with respect to the initial, energy-optimized structure. RMSD drift values were calculated from linear regressions. <R_G_> is the average radii of gyration during the trajectory. *R*_G_ error was lower than 0.001 Å in all p-HLA trajectories.

The radius of gyration (*R*_G_) of a bimolecular complex builds up as the partners constituting them dissociate. It also increases when a polymer like a protein unfolds. For each snapshot and any atom set, *R*_G_ was defined according to the following equation:

$$R_{G}=\sqrt{\frac{\sum_{i}m_{i}\cdot {\left|{\vec{r}}_{i}-{\vec{r}}_{CoM}\right|}^{2}}{\sum_{i}m_{i}}}$$ (1)

Wherein ${\vec{r}}_{i}$ and *m*_*i*_ account for the coordinates and mass of each atom of the set, respectively; and ${\vec{r}}_{CoM}$ is the coordinate of the center of mass (CoM) for the same set. Positions of the peptide CoMs relative to the main axes were determined using a modified version of the tk script previously described [46] in the console of VMD [47]. This script performs single value decompositions of the inertia moment matrixes of two atom sets to calculate their major rotation axes and, subsequently, provides a description of the relative orientation between them.

Salt bridges were defined by using a 8 Å distance cut-off between any sidechain carboxyl oxygen from an acidic residue *i* and the sidechain nitrogen from a basic residue *j*. To compare salt-bridge patterns we built a weighted adjacency matrix A, in which every element *a*_ij_ represented the average of the inverse of distances along the trajectory for a specific salt bridge. To compare two different matrixes with the same dimensions, A and B, the Frobenius’ distances were computed as follows.

$${\left\|A\right\|}_{FB}=\sqrt{Tr\left(A\cdot B^{H}\right)}$$ (2)

Where B^H^ is the conjugate transpose of B.

Conformational entropies were estimated by using the standard approaches implemented in the PTRAJ module of AMBER upon quasi-harmonic analyses of the mass weighted covariance matrixes for each system.

DelPhi v.5.1 [48] was used to analyze protein electrostatics by solving the Poisson-Boltzmann equation by a finite difference method. Protein dielectric constant value was set to 4 and grid size to 0.5 Å.

## Results

### Stability of simulations

Ten MD trajectories were set up to simulate the behavior of isolated components of the various peptide-HLA-TCR complexes known to show a high structural similarity (Fig 1) but a different functional behavior [22].

**Fig 2:**
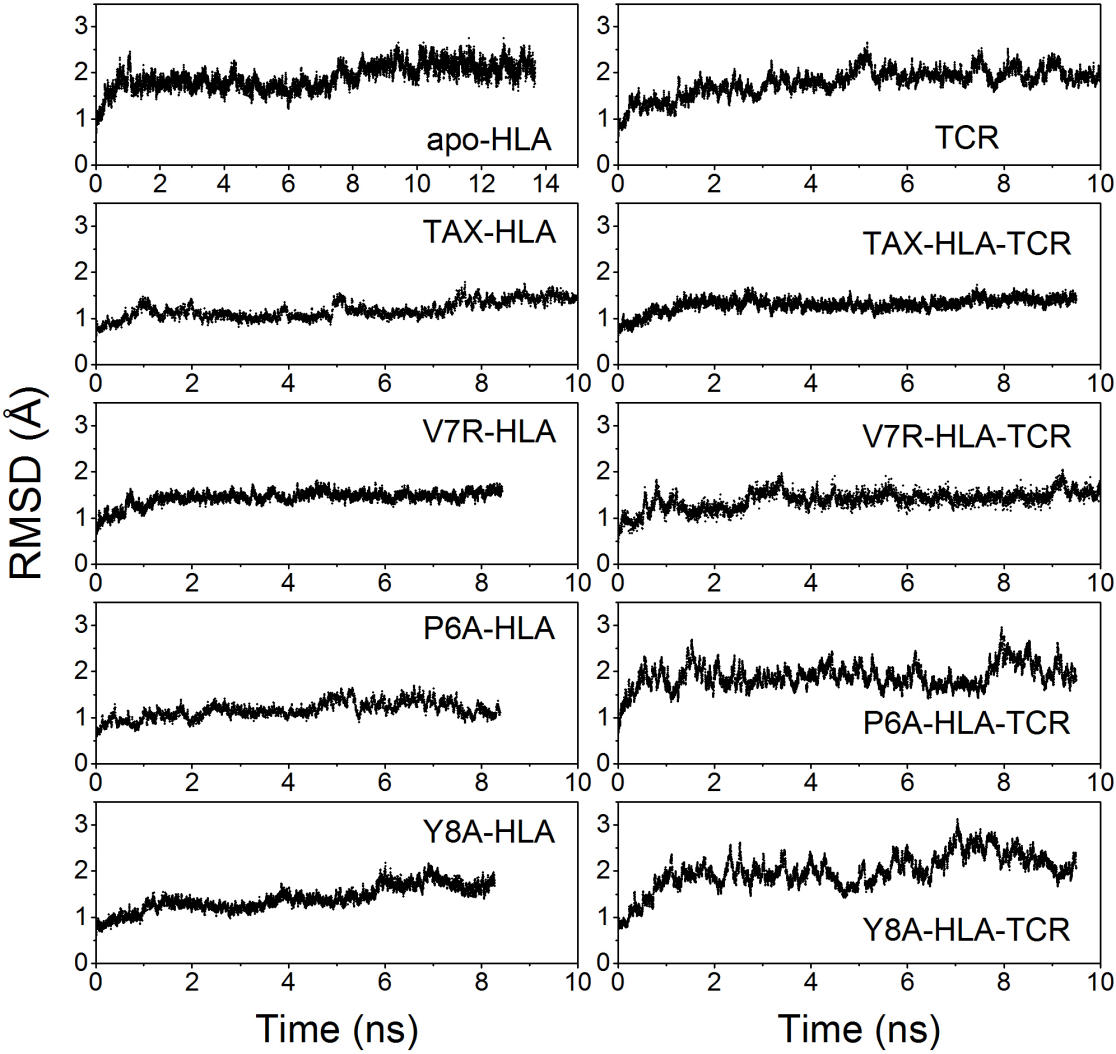
Time courses for the Root Mean Square Deviations along the simulations. RMSDs were calculated for the main-chain atoms, using the initial, energy minimized structure as reference. Further statistical data is summarized in Table 1.

Fig 2 shows the evolution of the structures along the trajectories, as displayed by the root mean-squared deviation (RMSD) values with respect to the initial structures. In agreement with the statistical data in Table 1, the structure of the distinct p-HLA secondary complexes barely changes along simulations. The average RMSD values are small for most of the binary p-HLA complexes indicating that their structure remains stable over the trajectories. The RMSD values observed for the ternary complexes are somewhat larger, but still small when considering that it is a complex involving 3 large polypeptides and the antigen oligopeptide.

Both, protein unfolding and complex dissociation are accompanied by an increase of the *R*_G_ values of the protein component of the simulated system. Hence, the radii of gyration (*R*_G_) were also monitored along the computations (Table 1). The initial *R*_G_ values for binary and ternary complexes were 17 Å and 30 Å, respectively. The absence of any increment or fluctuation in *R*_G_ values along the trajectories clearly indicates that the oligopeptides remain bonded to the HLA domain in all the calculations. Additionally, we monitored the position of the CoMs of the peptides using principal rotation axes of HLA as the reference coordinate system the (S1 Fig).

### The distinct p-HLA complexes are highly similar

Comparing the average structures of HLA bonded to reactive and non-reactive peptides reveals that they are highly similar. Their average all-atoms RMSD values relative to TAX-HLA (including sidechains) are 1.34 Å, 1.63 Å and 1.36 Å for V7R-HLA, P6A-HLA and Y8A-HLA, respectively. When the RMSD values are computed for every residue in the sequence, the only outlier from the statistical distribution corresponds to Asn86, located in the loop region following α-helix 1. This loop shows the highest mobility of the protein (see below).

### The helical regions of HLA are sensitive to the presence of the peptide

The somewhat larger RMSD values observed for the free HLA trajectory (2.65 Å on average) suggest that a small structural change takes place. In fact, the gyration radius of free HLA is smaller (15.22 Å) than those of the various p-HLA complexes (ca. 17.2 Å). Indeed, the α-helical regions of free HLA relax and approach each other due to the absence of the antigenic peptide in the cleft they define.

**Fig 3:**
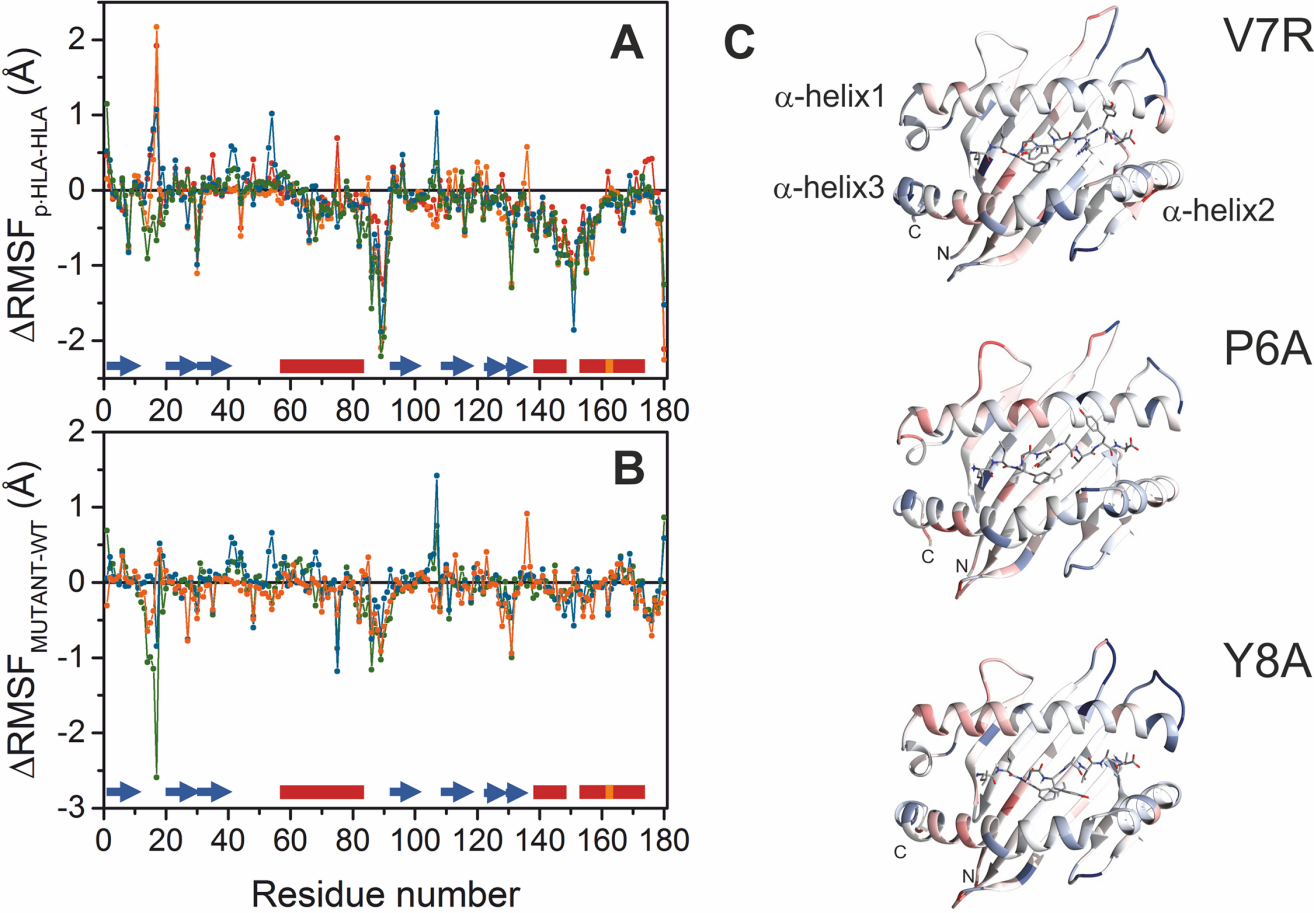
Root mean square fluctuations of HLA in the different complexes. (A) Differences between main-chain fluctuations of HLA in its free and binary forms, p-HLA. Data corresponding to TAX-HLA, V7R-HLA, P6A-HLA and Y8A-HLA are in red, orange, blue and green, respectively. Secondary structure elements are shown on top of the abscissa axis. Blue arrows represent β-strands and helical regions (α in red, π in orange) are shown as boxes. (B) Backbone fluctuations within V7R-HLA, P6A-HLA and Y8A-HLA complexes relative to the TAX-HLA complex. The N-terminus of the α-helix 1 and the central turns of the α-helix 3 present larger fluctuations (red) in the P6A-HLA and Y8A-HLA complexes than the reactive complex. (C) Maps of the main-chain RMSD differences between the mutant binary complexes and TAX-HLA. Colors scales from -1.2 Å (blue) to 1.5 Å (red).

The differences between the pattern of atomic fluctuations within HLA in its apo- and binary (p-HLA) forms are shown in Fig 3A. A positive value indicates a greater mobility of a residue in p-HLA with respect to free HLA. As expected, the values were negative in the α-helix regions that define the groove of HLA housing the antigen. Thus, their mobility decreases when they clamp the antigenic peptide. In particular, the fluctuations of α helices 2 and 3 resulted to be more sensitive to the presence of the antigen than α helix 1. In fact, the hinge between these two elements is highly restrained by the oligopeptide.

When checking the RMSFs of the binary p-HLA complexes, t-student tests showed no difference between TAX-HLA and V7R-HLA complexes. However, P6A-HLA and V8A-HLA showed a slight but significant increase of the average backbone fluctuations (Fig 3B), according to t-student tests at 5% confidence. In fact, although the pattern of fluctuations barely changed in the various p-HLA complexes, there were differences in specific residues related to the reactivity of the peptide bound to HLA (Fig 3B and 3C). For instance, the region comprising the amino acid at positions 38–48 and 100–110 fluctuated more in the non-reactive than in the reactive complexes. Notably, Trp107 shows the largest fluctuations in non-reactive P6A-HLA and Y8A-HLA. Trp107 sidechain is in close contact with α helix 3, which is involved in TCR binding. Nevertheless, the largest differences locate at loops that locate out of the surface patch of p-HLA that contacts TCR. Taking the wild-type TAX-HLA reactive complex as a reference, we examined the fluctuations of the non-reactive and weakly reactive V7R-HLA, P6A-HLA and Y8A-HLA complexes (Fig 3B and 3C). However, data reveals a slight enhancement of fluctuations of the N-terminus region of α-helix 1, whereas the C-terminus of α-helices 2 and 3, the hinge region between these two elements and Gly162 – which locates at the π-helix region in the middle of helix 3—decrease their mobility.

### Effects of TCR binding on p-HLA dynamics

Binding to the T-cell receptor (TCR) hardly affects the structure of p-HLA. In fact, the average RMSD of the binary reactive complex TAX-HLA is nearly identical (1.36 Å) to its average value computed in the ternary reactive complex TAX-HLA-TCR (1.33 Å). The average RMSD values of the non-reactive and weakly reactive complexes were also below 2 Å, though they were higher in the ternary complexes than in their respective binary forms.

To test the internal mobility within the various molecules, we analyzed the atomic fluctuations (RMSFs) of the main-chain atoms. The pattern of TCR fluctuations was very similar in the different complexes. Notably, p-HLA binding barely restrains the TCR motions besides those of the CDR3 loops (S2 Fig) responsible for the molecular recognition of the p-HLA molecules, and some flexible loops located at the constant α and β domains. We also analyzed the fluctuations in the apo and antigen-bonded HLA species (S3 Fig).

Upon binding TCR, HLA displays an overall drop in its backbone fluctuations (S3 Fig), according to t-student tests at 1% significance–*p* values being lower than 10^−8^. In addition, a specific loss of mobility was found for the unreactive p-HLA complexes in the stretch comprising amino acids 85 to 92, which corresponds to the loop joining α-helix 1 to β-strand 4.

Hence, to investigate to which extent the differences in the atomic fluctuations may affect the binding equilibrium (Fig 3B and text above), we calculated the contribution of conformational (vibration) entropy (ΔS^conf^) of free TCR, binary and ternary complexes and then of the association following the cycle ΔS^conf^_ternary_− ΔS^conf^_freeTCR_− ΔS^conf^_binary_. Data in Table 2, computed using all the protein atoms, indicate that vibrational entropy contribution is less favorable for the formation of ternary complexes involving non-reactive antigens, in agreement with the slightly higher fluctuations found in their binary complexes. In other words, these differences in conformational entropy are related to the higher p-HLA entropy in the free non-reactive p-HLA complexes and the smaller entropies found in the non-reactive ternary ones. The same trend is observed when only backbone atoms are considered, though the differences are much smaller, in agreement with the tiny, but significant differences found in backbone RMSFs (Fig 3B). Hence we attribute most of the changes in the vibrational term of the binding entropy to the changes in sidechain dynamics.

**Table 2:** Vibrational entropy contributions (-TΔS^conf^) to binding-free energy.

	-TΔS^conf^_p-HLA_	-TΔΔS^conf^_p-HLA-TAX-HLA_	-TΔS^conf^_ternary_	-TΔS^conf^_binding_	-TΔΔS^conf^_binding_
TAX	-9905.03	0.00	-30625.79	2828.31	0.00
V7R	-10314.69	-409.66	-30506.61	3357.16	528.84
P6A	-11396.40	-1491.37	-30763.30	4182.17	1353.86
Y8A	-11319.11	-1414.08	-30407.24	4460.94	1632.62

Units are kJ/mol. ΔS^conf^ stands for the vibrational entropy as computed by the PTRAJ module of AMBER. Ternary, p-HLA-TCR complexes. **-**TΔΔS^conf^ (kJ/mol) was calculated in each case with respect to TAX values. -TΔS^conf^ for free TCR was estimated to be -23549.07 kJ/mol.

Salt-bridge patterns and electrostatics in reactive and unreactive complexes

**Fig 4:**
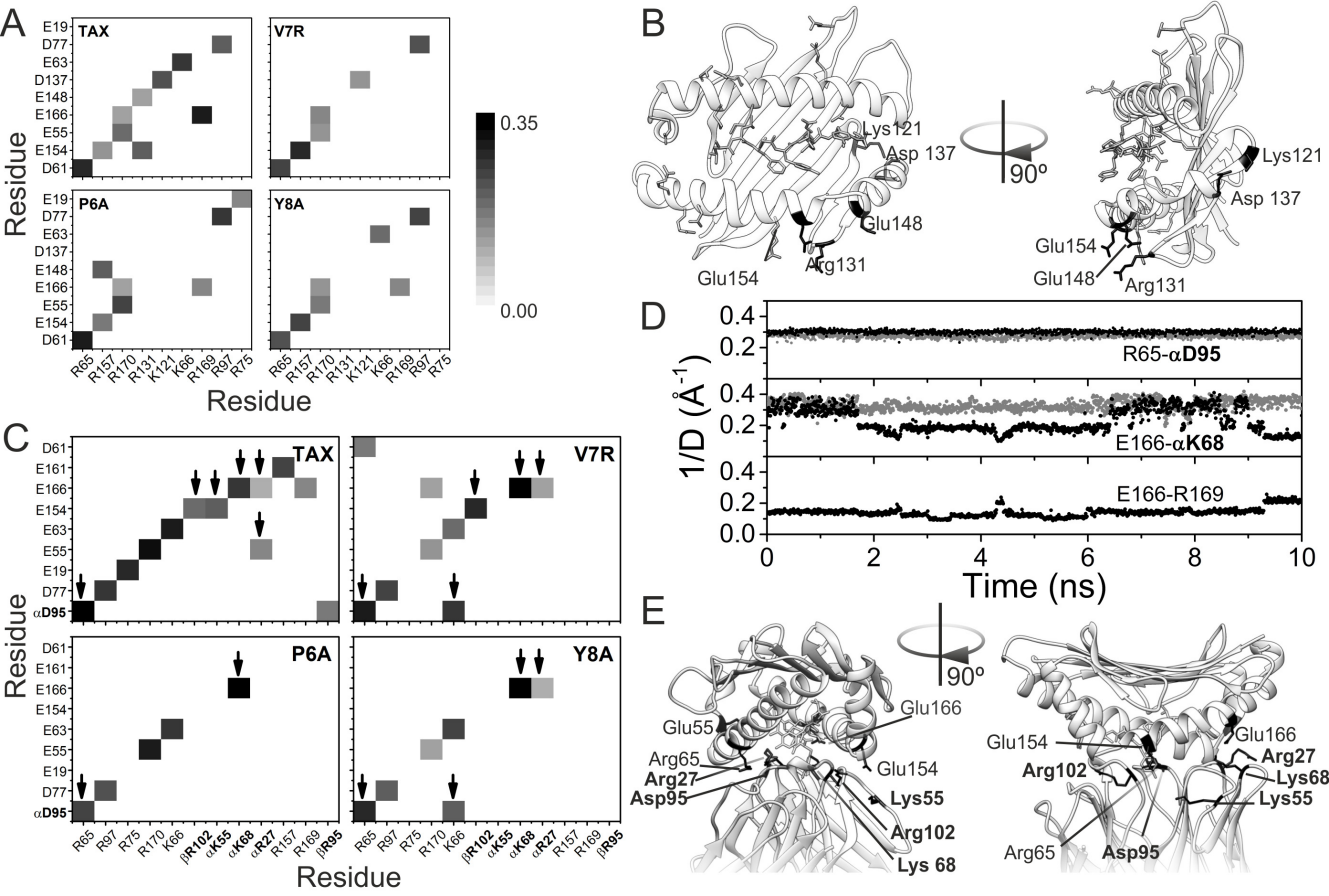
Salt bridge patterns. (A) Patterns of salt bridges in the binary complexes, p-HLA. The normalized weight (proportional to the inverse of the distance between the heavy atoms of the two charged groups) of each salt bridge is represented as a grey scale from 0 (white) to 0.35 (black). (B) Representation of the TAX-HLA binary complex structure. Residues involved in salt bridges are represented in sticks. Labeled, black-colored residues are involved in salt bridges that are lost in non-reactive p-HLA complexes. (C) Pattern of salt bridges in the ternary complex, p-HLA-TCR, colored as in (A). TCR residues are in bold font, and with a Greek letters indicating the chain to which the residue belongs. Arrows point to HLA-TCR intermolecular bridges. (D) Time evolution of different salt bridges detected in the ternary complexes involving TAX (black dots) and the Y8A mutant (grey dots). The ordinate axis corresponds to the inverse of the distance between heavy atoms of the two residues, to allow comparison with panels A and C. (E) Detail of the structure of the TAX ternary complex (pdb code: 1ao7), displaying in sticks the antigen (in white) as well as the residues involved in intermolecular HLA-TCR salt bridges (in black). Again, bold font labels correspond to residues from TCR, plain font ones to HLA.

The salt bridges were studied in all the trajectories of the different binary and ternary complexes (Fig 4), considering ion-pairs with an oxygen-nitrogen distance lower than 3.2 Å in the peptide-HLA-TCR interface.

With respect to the p-HLA binary complexes, we observed ten salt bridges in TAX-HLA, six in V7R-HLA, eight in P6A-HLA and seven in Y8A-HLA (Fig 4A). These salt bridges are intramolecular interactions between charged groups of HLA. The electrostatic potential energy for a given pair of charges is inversely proportional to the distance between them, so we calculated the average of the inverse of the distances of every salt bridge during the trajectory of the complexes. The resulting data was represented as a matrix for the four p-HLA complexes. The observed patterns in these matrices varied between them. Notably, the weakly reactive and non-reactive complexes had fewer salt bridges than the reactive one. The pattern of the weakly reactive complex (V7R-HLA) is intermediate between the patterns of the reactive (TAX-HLA) and non-reactive (P6A-HLA, Y8A-HLA) complexes. In fact, five salt bridges were common to all complexes (Asp61-Arg65, Glu154-Arg157, Glu55-Arg170, Glu166-Arg170 and Asp77-Arg97). In contrast, we noted the absence of three salt bridges from the non-reactive complexes (Glu148-Arg131, Glu154-Arg131 and Asp137-Lys121) that were present in the reactive complex (TAX-HLA). These salt bridges involve residues from α helices 2 and 3, and the rim of the β-sheet (Fig 4B). They may restrain the motion of these two helices in the reactive complexes, thereby favoring the interaction with TCR by decreasing the entropy loss during binding. Notably, Glu154 participates in salt bridges within the ternary complexes whereas Lys121, Arg131, Asp137 and Glu148 do not.

In conclusion, we observed a signature salt bridge pattern in the peptide-HLA complexes that varied according to the reactivity of the complex. This pattern is consistent with the distinct backbone fluctuation profiles.

We next analysed the salt bridges formed at the p-HLA-TCR interface (Fig 4C). As observed within the binary p-HLA complexes, we noted a significant loss of salt bridges in the non-reactive ternary complexes relative to the reactive complex. In particular, the reactive complex TAX-HLA-TCR had thirteen salt bridges; the intermediate complex V7R-HLA-TCR had ten, and the non-reactive complexes P6A-HLA-TCR and Y8A-HLA-TCR had five and seven salt bridges, respectively. Accordingly, the number of intermolecular salt bridges between p-HLA and TCR was also lower. The reactive complex had six intermolecular salt bridges whereas the intermediate complex had five and the non-reactive complexes P6A-HLA-TCR and Y8A-HLA-TCR had two and four intermolecular salt bridges, respectively. This may explain in part why the interaction between HLA and TCR is weaker in these complexes than in the reactive ones. The strongest interactions, like the bridge between Arg65 in HLA and Asp95 in TCR remain stable along the full simulation (Fig 4D). Interestingly, the intermolecular bridge between Glu166 in HLA and K68 in TCR is weaker in the native complex, and it seems to be modulated by the trend of Glu166 to interact, though more weakly, with Arg169.

We noted an absence of seven salt bridges in the feebly reactive and non-reactive complexes (Fig 4C). Three of these are intermolecular between HLA and TCR (Glu154 of HLA with Arg102 of the chain β of TCR, Glu154 of HLA with Lys55 of chain α of TCR, Glu55 of HLA with Arg27 of chain α of TCR). The two first are lost in the non-reactive complexes: those involving P6A and Y8A. Three salt bridges modulated the mobility of α helix 3 of HLA (Glu19-Arg75, Glu161-Arg157 and Glu166-Arg169). This may account also for the differences in vibrational entropies mentioned above. In addition, we found five salt bridges to be present in all complexes (Asp77 with Arg97 of HLA, Glu55 with Arg170 of HLA, Glu63 with Lys66 of HLA, Arg65 of HLA with Asp95 of the α-chain of TCR and Glu166 of HLA with Lys68 of the α-chain of TCR), thus suggesting them to be conserved and well-organized. In the V7R-HLA-TCR complex, six salt bridges were lost, whereas three new ones were gained. One of them involves residues from both HLA (Lys66) and TCR (Asp95). In the Y8A-HLA-TCR complex, only one new salt bridge was gained, and this was the same as that observed in the V7R-HLA-TCR complex (Lys66 of HLA with Asp95 of the α chain of TCR).

In conclusion, we observed a signature salt bridge pattern in the ternary peptide-HLA-TCR complexes that differed in its reactive complex compared with weakly reactive and non-reactive ones.

To quantify these salt bridge signatures, we calculated the Frobenius’ distances between the matrixes representing the distinct patterns (Table 3). This parameter measures how different two matrices of the same dimensions are. The larger the distance is, the more different are the two compared matrixes. Clearly, non-reactive ternary complexes are more similar to each other with respect their salt bridge pattern than they are to the reactive form. This trend is less pronounced in the binary complexes and more noticeable when only the intermolecular HLA-TCR salt bridges are considered.

**Table 3:** Normalized Frobenius’ distances between salt bridge matrices.

	TAX	V7R	P6A	Y8A	Y8A*
Y8A	0.34	0.25	0.31	0	0
P6A	0.46	0.34	0	**0.29**	**0.11**
V7R	0.44	0	**0.46**	**0.34**	**0.31**
TAX	0	**0.59**	**0.51**	**0.57**	**0.32**

Binary and ternary complexes are indicated by normal and bold type, respectively.

*Computed using intermolecular salt bridges matrixes.

**Fig 5:**
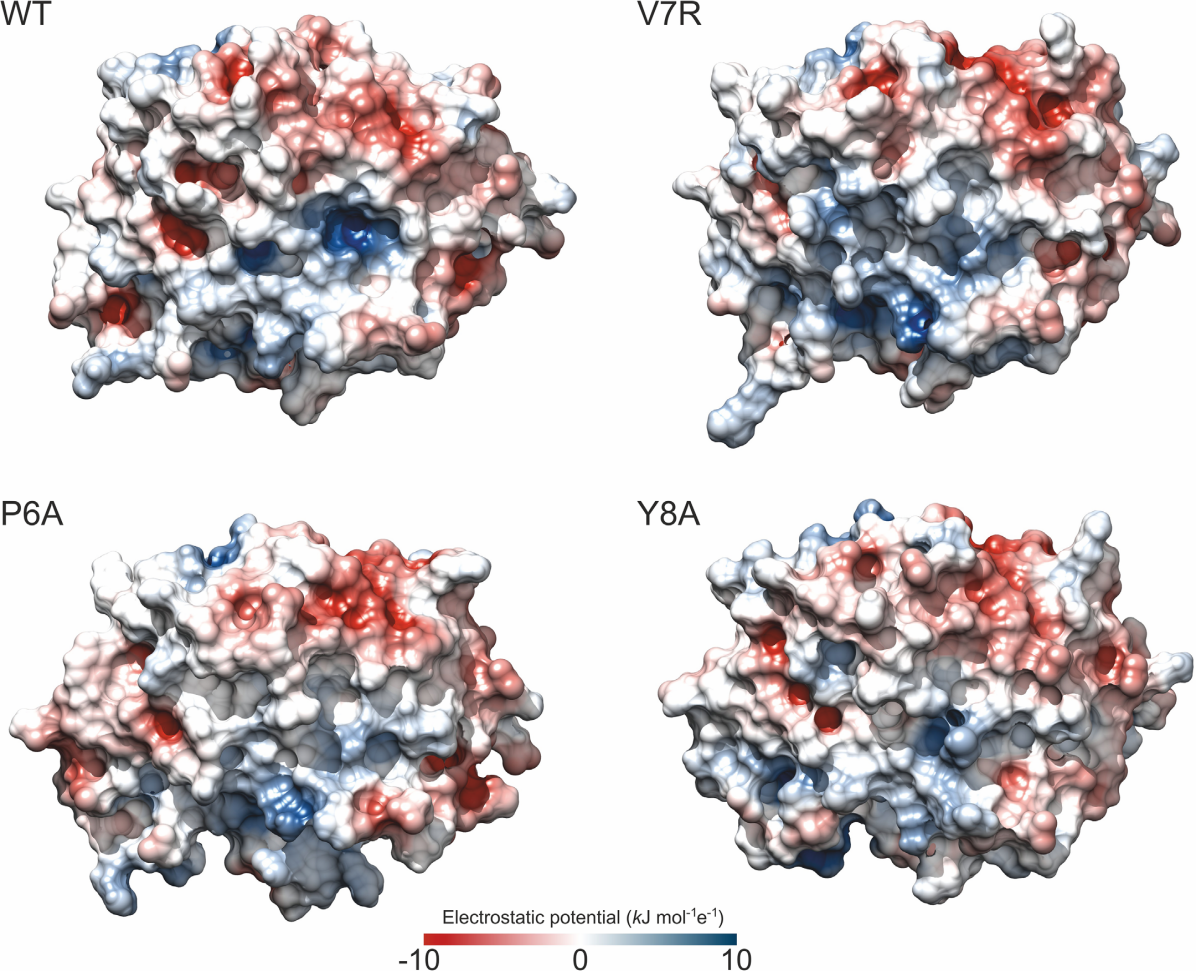
Electrostatics of the p-HLA binary complexes. Electrostatic potential maps at the surface of WT and mutant p-HLA binary complexes. Color scales from dark blue (10 kJ mol^-1^ e^-1^) to dark red (-10 kJ mol^-1^ e^-1^); values at the surface lay in the range from -5 to 5 kJ mol^-1^ e^-1^. The electrostatic grids were calculated at 100 mM ionic strength using DelPhi software [48] and PQR files containing AMBER 2003 charges [41], as reported in Materials and Methods. Protein dielectric constant was set to 4.

We next studied how the change in the salt bridges affects the electrostatics of the various binary complexes (Fig 5). A single amino acid substitution in a peptide affects the electrostatic potential of the complex even if it does not modify the charge of the peptide, as is the case with the P6A-HLA and Y8A-HLA complexes. As the oligopeptide is held in a cleft, the mutations may affect p-HLA solvation and the boundary between two phases with different dielectric constants: the protein and the solvent. In its turn, these changes may affect how the charged residues interact at the protein surface. Nevertheless, in the complex V7R-HLA, the substitution modifies the charge, since a non-polar amino acid (valine) is replaced by a polar amino acid (arginine) at position seven. We observed that positive regions were strongly attenuated by this substitution. Surprisingly, this also occurred in the non-reactive complexes, even without a change of charge. In the V7R-HLA complex, however, this local attenuation was accompanied by an expansion of the positive potential through the HLA cleft region that was not seen in the case of the neutral mutants.

## Discussion

The structures of the p-HLA-TCR complexes are somewhat flexible, as becomes more evident as new structures are resolved [49,50]. Hence, we have analyzed the dynamics of various binary complexes, and their corresponding ternary ones (p-HLA-TCR), with a different degree of immune reactivity in order to clarify the molecular cause of the range of T cell response patterns seen. The Tax/HLA-A0201 was chosen as a model system because it has been extensively studied experimentally. Ding *et al*. were one of the first groups to study these peptides in the context of different T cell signals [9,22]. They calculated the binding thermodynamics and analyzed the conformational changes of the three structures of HLA-A2/TCR-A6 complexes containing singly substituted variants of the reactive peptide TAX. Their work provided direct evidence that p-HLA-TCR complexes capable of generating extremely different signals have very similar structures. The capacity of variant TAX peptides (V7R, P6A and Y8A) to produce different biological effects upon interaction with the TCR-A6 was demonstrated with T cell assays in which the specific lysis, secretion of interferon- γ (INF-γ) and macrophage inflammatory protein 1β (MIP-1β) were measured. They confirmed that the V7R peptide, but not the P6A or Y8A peptides, is able to functionally engage the TCR-A6. We chose the TAX peptide and its mutants as a model system because the pattern of immune reactivity and affinity for TCR is well established. Ding *et al*. [9] also described two TCRs (TCR-B7 and TCR-A6) that bind the same p-HLA complex, giving rise to the TAX/HLA-A2/TCR-A6 and TAX/HLA-A2/TCR-B7 complexes. Both p-HLA-TCR complexes initiate the same strong agonist signal. In summary, the complexes herein analyzed display very similar conformations but different functional behavior. The complex between TAX-HLA and TCR shows a dissociation constant in the micromolar range, V7R-TAX shows a ten-fold decrease in the affinity and, finally, P6A and Y8A complexes are too weak to be analyzed by biophysical methods like SPR [22]. Hence, protein dynamics may take part in the recognition, as observed by infrared spectroscopy in other systems [27, 28].

For decades, the study of assembly mechanisms and immunological responses has focused on TCRs and their conformational changes [51–53]. In the current study, we calculated the RMSD of all ternary complexes and concluded that TCR recognition domains are well superimposed. Conversely, p-HLA displayed substantial differences in its dynamics when the free and ternary (p-HLA-TCR) forms were compared. Our study suggests that p-HLA dynamics plays a major active role in T cell activation. The pattern of salt bridges between the peptides and the HLA varies depending on the oligopeptide. For the free p-HLA species similarity between these patterns–according to Frobenius’ distances–seem not to correlate with reactivity. However, the lack of specific salt bridges may be responsible for the changes in the electrostatic properties and the increase in vibrational entropy in the unreactive p-HLA complexes. Notably, although this parameter does not account for overall binding energy–entropy is not an additive property, and the binding energy is a residual of various contributions with different signs—it correlates with the distinct reactivities of the p-HLA. This conclusion is in agreement with the experimental results obtained by infrared spectroscopy in other systems [27,28]. This is also consistent with the previous report by Borbulevych *et al*. [54] showing that an enhanced dynamics Tel1p-HLA-A2 with respect Tax-HLA-A2 is responsible for their distinct abilities to interact with TCR molecules. Thus, differential tuning of the dynamic properties of HLA-A2 by the Tax and Tel1p peptides facilitates cross-recognition and affects the way structural diversity can be presented to and accommodated by immune system receptors [54]. Accordingly, a single HLA polymorphism alters the dynamics of the p-HLA landscape and affects TCR recognition. The micro-polymorphism in the HLA-B44 allotypes alters the mode of binding and dynamics of the bound viral epitope [28]. Other studies defend the role of peptide flexibility of the assembly mechanism of the p-HLA-TCR complexes [55–57].

Our study shows significant changes of salt bridges in the binary and ternary complexes of non-reactive complexes relative to the reactive ones. As shown in Fig 5, loss in non-reactive binary complexes affects the electrostatic potential. This change in the electrostatic component probably contributes to the differences in total free energy between complexes reported by other authors [33,58]. Additionally, three of the missing bridges restraint the motion of the helices, so their loss could be responsible for the increase in conformation entropy for the free p-HLA form. On the other hand, five salt bridges are found in all p-HLA and p-HLA-TCR complexes. These conserved salt bridges may be involved in the stability of the interaction of p-HLA in the binary complex and in the stability of the ternary form. These findings are consistent with the recent work of Xia *et al*. [59], who also found three common salt bridges in the HLA B*2705-KK10 system and the clonotypes TCR-B3, TCR-B5 and TCR-B6. This signature salt bridge is conserved in all complexes. Its disruption by site-directed mutagenesis results in conformational distortions and a low affinity towards TCR [59]. Nevertheless, the strength of these conserved bridges may be modulated by the presence of nearby residues.

As expected, the Frobenius’ distances for the salt bridge patterns in the ternary complexes correlate with reactivity. In the crystal structure, we found four salt bridges in the reactive and intermediate complexes and three in the non-reactive complexes. In all of them, three of the salt bridges observed in the crystal structure (Asp77 with Arg97 of HLA, Arg65 of HLA with Asp95 of TCR, and Glu166 of HLA with Lys68 of TCR) were kept along the trajectories, except for the non-reactive complex P6A-HLA-TCR, for which only two salt bridges matched. Moreover, the interaction between Arg65 of HLA with Asp95 of TCR resulted to be less stable in the reactive complex, most probably because in the corresponding binary complex, Arg169 competes for the interaction with Glu166 more than in the non-reactive ones. These differences arise from the experimental crystallographic data usually being recorded at a low temperature (77 K), thereby reducing the dynamics of the protein. A similar example is found in electron transfer complexes, for which the observation of specific salt bridges through molecular dynamic computations [46,60] explained the discrepancies between kinetic analyses of site-directed mutants [61] and structural data [62].

To summarize, molecular dynamics enabled us to discern patterns of salt bridges, in p-HLA and p-HLA-TCR complexes that differ according to the reactivity of the complex. In the binary complexes, salt bridges may affect their affinity towards TCR by modulating protein mobility and the subsequent entropy loss during the formation of the ternary complex. Our results are consistent with the work of Hawse *et al*. [63], who argued that tuning HLA dynamics constitutes a fundamental mechanism for modulating antigenicity. Taken together, our findings prompt us to conclude that modulation of p-HLA dynamics plays a major role in T cell activation. Of particular relevance is that TAX variants modulate in a different degree the dynamics of surface sidechains in HLA to strongly affect its affinity towards TCR.

## Supporting Information

S1 Fig Coordinates of the Centers of Mass of TAX peptides relative to the main rotation axes of HLA along the trajectories of the binary complexes. A) Cα carbon traces of HLA (grey) and TAX (black) are represented together with their respective CoMs and principal rotation axes, computed as previously reported in other systems [46]. Rotation of axis in cyan (X)—the shortest one—displays the maximum orthogonal inertia moment component. B) Projections or the position of the CoM of the peptide on the XZ and XY planes defined by the main axes of HLA. Left panels show the data corresponding to wild-type TAX (in red), and right panels displays those for V7R (orange), P6A (blue) and Y8A (green).
(TIF)

S2 Fig Analysis of RMSFs of TCR in the different complexes. The Fig represents the differences in atomic fluctuations between bound and free TCR. The value of zero on the ordinate axis corresponds to the fluctuations of free TCR. The top and bottom panels show α and β chains of TCR, respectively.
(TIF)

S3 Fig The fluctuation pattern of p-HLAs in their binary and ternary forms. (A), main chain root mean square fluctuations for apo-HLA (black), TAX-HLA (red), V7R-HLA (orange), P6A-HLA (blue) and Y8A-HLA (green). (B), root mean square fluctuations of main chain atoms for the ternary complexes, colored as in (A). (C) Differences between the RMSF values of HLA main chain atoms in binary and ternary complexes, colored as in (A).
(TIF)
