# Supplementary figures and images for: The Dynamics of the Human Leukocyte Antigen Head Domain Modulates Its Recognition by the T-Cell Receptor
Source: PLoS One. 2016 Apr 28;11(4):e0154219. doi: 10.1371/journal.pone.0154219 (PMC4849770; doi:10.1371/journal.pone.0154219)

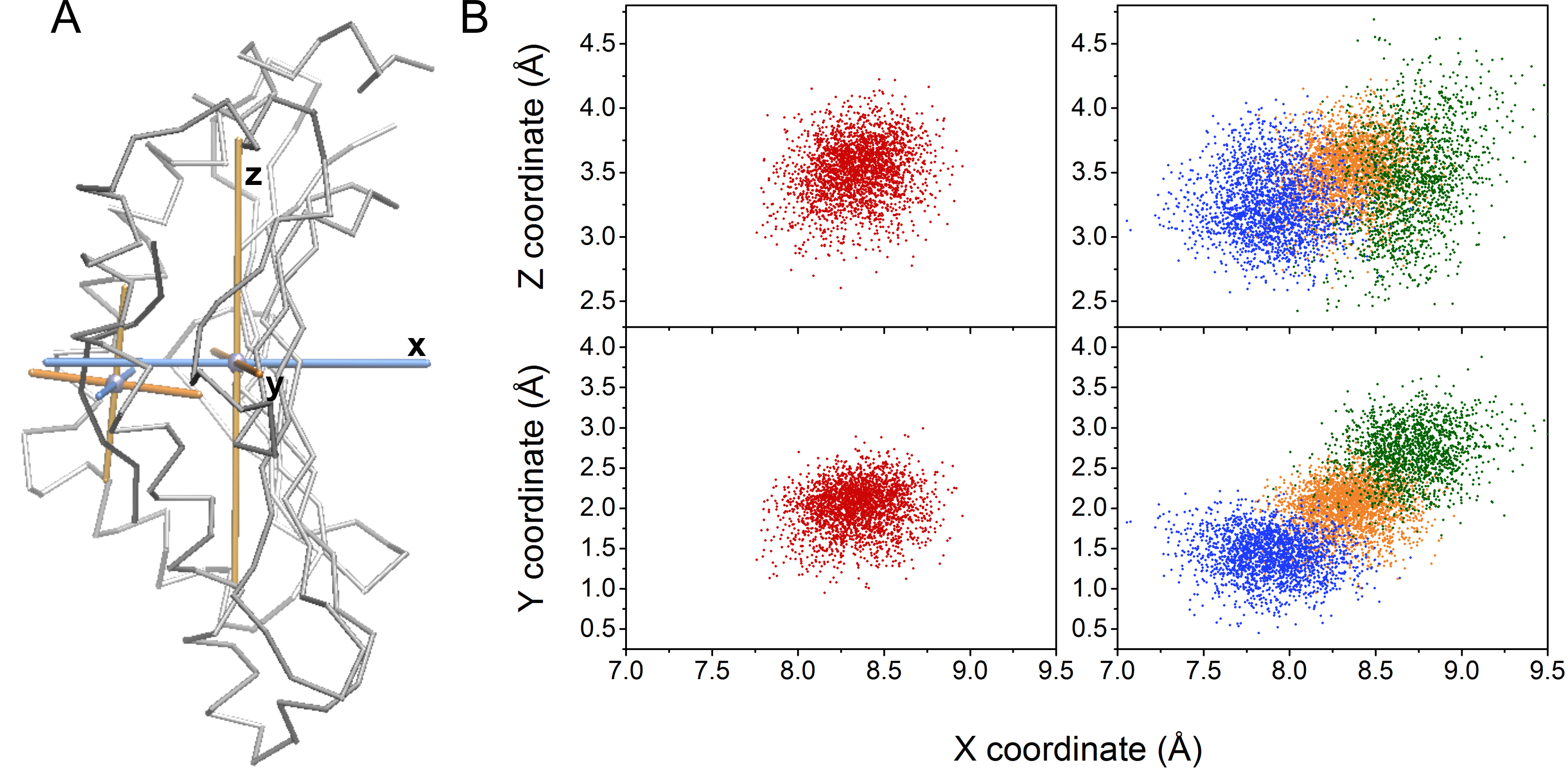

Supplement: S1 Fig — A) Cα carbon traces of HLA (grey) and TAX (black) are represented together with their respective CoMs and principal rotation axes, computed as previously reported in other systems [46]. Rotation of axis in cyan (X)—the shortest one—displays the maximum orthogonal inertia moment component. B) Projections or the position of the CoM of the peptide on the XZ and XY planes defined by the main axes of HLA. Left panels show the data corresponding to wild-type TAX (in red), and right panels displays those for V7R (orange), P6A (blue) and Y8A (green). (TIF) [file pone.0154219.s001.tif]

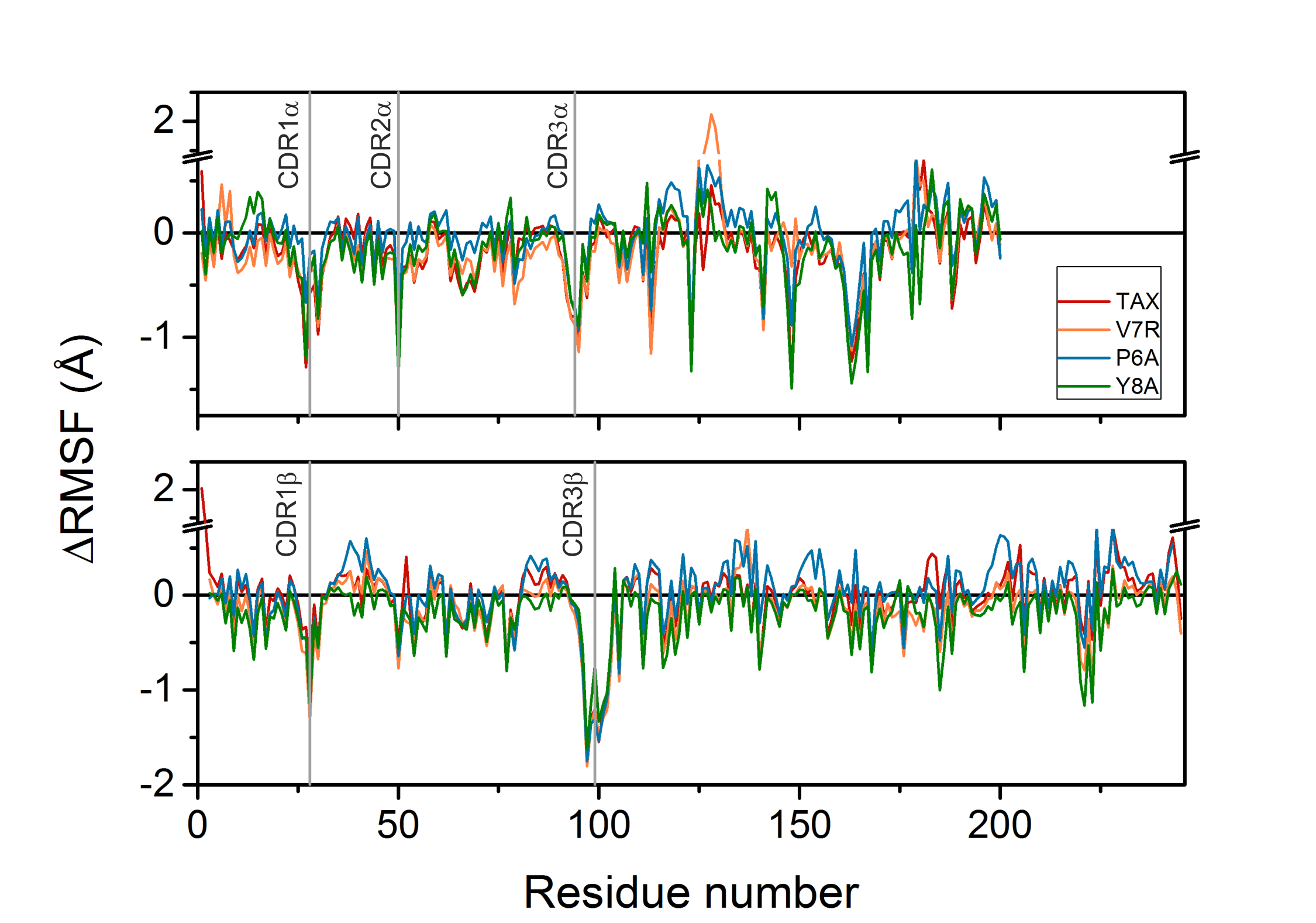

Supplement: S2 Fig — The Fig represents the differences in atomic fluctuations between bound and free TCR. The value of zero on the ordinate axis corresponds to the fluctuations of free TCR. The top and bottom panels show α and β chains of TCR, respectively. (TIF) [file pone.0154219.s002.tif]

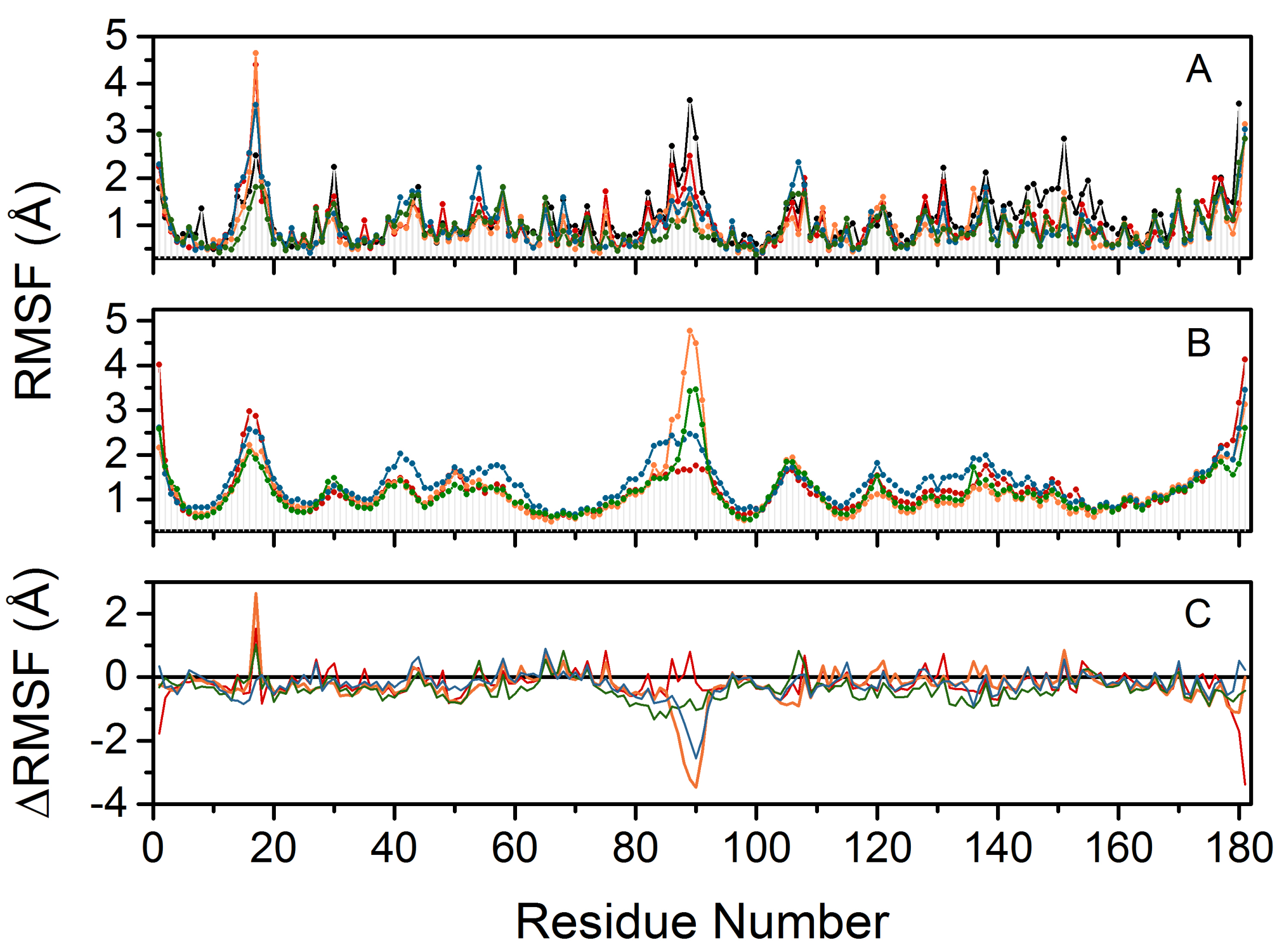

Supplement: S3 Fig — (A), main chain root mean square fluctuations for apo-HLA (black), TAX-HLA (red), V7R-HLA (orange), P6A-HLA (blue) and Y8A-HLA (green). (B), root mean square fluctuations of main chain atoms for the ternary complexes, colored as in (A). (C) Differences between the RMSF values of HLA main chain atoms in binary and ternary complexes, colored as in (A). (TIF) [file pone.0154219.s003.tif]
